# An Address

**Published:** 1859-03

**Authors:** W. W. Allport


### AN ADDRESS,

To the Graduates of the Ohio College of Dental Surgery, Session of 1858–59.

BY W. W. ALLPORT, D.D.S.

GENTLEMEN:—In what I have to say this evening, I desire not so much to amuse you with the flowers of Rhetoric, as to say a few plain and earnest words, which will be of use to you as you go out from this hall to engage in the duties of life.

Your preparatory course has ended, and your professional life is begun.

A vigorous and systematic course of preparatory training is requisite for success in any honorable calling. You have had the privilege of thus preparing yourselves for the Dental profession; and it will always be a pleasure for you to look back upon the thorough training you have here received, unless you have wasted these advantages by your own neglect. If you have had no higher ambition than to pass the examination of this College, and see your names ornamented with the title of D. D. S., you might have spared yourselves both the anxiety and expense; for unless you deserve it, the title, in the end, will be useless. It may serve you as an introduction—very likely it will to some extent—but so far as it does, the world will expect that the title represents skill, and that you are what you seem to be.

False colors in a profession, like the " ALIAS " of a rogue, may serve as a temporary shift for dishonesty; but in the end it will bring disgrace. As in the fable, the ass in the lion's skin is revealed by his bray. In this country, titles amount to very little. The people, to get rid of a brawling demagogue, may send him to the legislature; but his "*rising in his seat*," or having "*Hon.*" printed before his name, does not make him a statesman.

Such men as Jefferson, Clay, Calhoun and Webster made the title of "Honorable," really of *honorable* distinction; but it has been so often disgraced, that now it may be more than doubted whether it is a higher title than the plain English "*Mr.*" The community care but very little whether a name begins with *Hon.* or ends with Esq.

A medical College may, for a *consideration*, make an M. D.; but no college can make a Mott, a Brainard, or a Hamilton. The M. D. will make a "*Doctor*," and four 'bi ts" invested in the skill of a painter, will do the same; but neither will make the *true* physician or surgeon.

Some rich congregation may secure a D. D. for a dull pastor, but

> "The pith o' sense and pride o' worth,
> Are higher ranks than a' that."

and the people will be glad to exchange preacher, doctor and all, for the brains of a Beecher, a Barnes, or a Schyler. Titles are often purcharsed, and sometimes stolen. Small men, like small inns, are apt to thus assume large names. There is hardly a log city in the country without its "Astor," or "Girard House," or a "United States Hotel," a story and a half high; but the names do not make the inns more inviting; and he who trusts to the large name of a public house, as a guaranty of comfort, or to titles as an indication of a man's ability, will be very apt to receive *bites* not indicated by the titles, or advertised in the "bill of fare." I trust, therefore, it has not been for the title, but for the *instruction*, that you have attended this College.

The title of Doctor has been supposed to represent learning and skill in the healing art; but it has been so largely appropriated by itinerant lecturers, horse doctors, and the unlettered in science generally; and Medical Colleges have made so many "Doctors," where they had not the *material* to make *Physicians*, that it now really means only that a dabbler in medicine has hired an office, and "hung out his shingle."

The quacks in our profession usually write Dr. before their names; and I am sorry to say, in the rivalry to graduate a a large number of students, our Dental Colleges have, in some cases, been induced to follow too nearly the example of our mother profession, and have allowed unworthy persons to write that which was *designed* as a title of merit, after their names. The title you have just received is so little understood by the public, that if you use it, it will require the word "Dentist," to explain its meaning; and you will agree with me, that it would not be in as good taste for even Maynard, Taylor or Dunning, to display his title of D. D. S. on his sign, as the plain word Dentist. Some of our profession seem to think that dentist is not respectable enough. This, to say the least of it, is a foolish weakness; and, may I not add that in a majority of such cases, (in even their own estimation,) the title they would assume would be the most *attractive* portion of their names.

If it were proper, I could mention scores of men, whose abilities, both natural and acquired, would adorn and enoble any profession; and yet they ask no other title than Dentist. It is by this title that you will be known. If it is not of equal respectability to that of any other, certain it is that it is not because ours is not a useful profession, but on account of those who practice it; and it is your duty to help to make it of equal respectability to that of any other. The only value of your title is to tell your calling, and if you honor it, as I hope you will, you need ask nothing more, and the people will apply the title of Doctor to you, to your heart's content. For this reason, I advise you to tell your painter to make you simply—"JOHN SMITH, DENTIST."

When your location has been chosen, it is not likely that your sign will attract a crowd at first. The sign only tells where John Smith, Dentist, lives. Who *Smith* is, few know, and *fewer* care. As yet, John Smith is only a member of the "Smith family." The glitter of the new sign may attract some who are out *shopping*, for "sets of teeth ready made," or who desire to know where dentistry is done "to order," on the shortest notice, and "*most reasonable terms.*" But the thinking and paying portion of a community will not be in a hurry to believe John Smith, the new dentist, to be the greatest man in the world, until they know something about him. They intend to find out who he is, what he knows, and what he can do, before they give him much employment. They will ask a hundred questions about him, of which he never dreams. Is he a man of *character?* Is he intelligent, honest, and skillful in his profession? When these, and similar questions, are answered favorably, they will trust him, and put their wives and daughters under his care. When he has *merited* a good character, he will *have it;* and John Smith, *Dentist*, will be an institution, and be regarded as one of the necessary workers of society; and success will not depend so much upon the gilded and *ornamented* "John Smith," that swings on the outside of the office, as upon the *honest* and *skillful* John Smith, who works within.

The first patient will be an important event in your professional life. Do not think it makes a man wise to put on wise *looks*. You will never make a greater mistake than to suppose that you can impress *sensible* people with the idea that you are a great man, by putting on *airs*. You should not assume knowledge you do not possess; neither should you allow a lack of independence to prevent you from impressing your patient with the knowledge you do *really* possess of your profession. Your best way will be, to express your thoughts, and answer plain questions, in plain words.

It is to be presumed, if you do not know to the contrary, that your patients speak the English language, and will understand it at least as will as French or Latin.

When patients come to us for counsel, they desire to learn facts, and not to take lessons in the languages, or in the glossary of dentistry. Our profession is in its nature such, that almost every patient can be made to understand its principles, if we use plain English to explain it to them; and as our object *ought* to be, to instruct our patients, we should speak to them in language they can understand. This you may think of very little importance; but in some cases, you will find, that it will make the difference between business gained and business lost. My advice to you therefore is, to speak your mother tongue.

Your office should be cheerful; for a cheerful room makes a great difference to a sufferer. Your furniture should be neat, carefully selected,—enough for use, and too little for display. It ought to be equally removed from the profusion of a furniture ware-room, and the barrenness of a miser's apartments. It will not add to the attraction of your office to make it the fac simile of a bar-room, with the vile smell of cigars and tobacco; nor will the recollection of it be more pleasant, if it be uncleanly and in disorder.

In this age of extravagance, you may be tempted to believe that rosewood and damask are patents of nobility. You will find, however, that your office will be furnished better, whether it be cheaply or expensively, when it speaks to every one that its occupant is a gentleman of taste. There is a quiet air of confidence inspired by neatness, and no heraldry so proclaims the gentleman as a well-ordered dress. Few things are more civilizing than cleanliness. Outcasts seldom indulge in the luxury of soap and clean linen. In some cases, it is true, the *hand* would be the thing contaminated, yet the Dentist who neglects cleanliness, and expects to thrust his dirty hands into a lady's mouth, should be regarded as unworthy of decent society.

You should endeavor to prevent your office from becoming the resort of loungers. There are few things more embarrassing to a delicate woman, than to meet, face to face, in the consulting room of the Dentist, half a dozen loungers, whose idleness must make them professional gossips. Some patients are very sensitive to any defects or decay which speak of the inroads of disease, or time; and you can not more effectually drive such patients from you, than to let them know that your office is the idlers' lounging place. You should remember that your relations with your patients are confidential, and there can be no apology for revealing them yourselves, or allowing those who will, about you. Promptness and industry on your part, during office hours, and strict attention to your professional duties, will free you from the annoyance of these idlers, who are gossips because they have nothing else to do. With promptness, on your part, to meet your engagements with your patients, you have the right, which you should *insist upon*, to demand promptness on their part. This will save the time of all and give the business man confidence in you as one who looks after the details of his business; and will also prevent conflict in your appointments.

You should provide yourselves with a register of convenient form, in which you can keep a correct record of all your operations, and write out an accurate description of all peculiar cases; this will frequently prevent misunderstanding with your patients, in regard to the failure of your work, and will also, in a few years, furnish you with a series of facts, which otherwise would have been forgotten, from which you can draw accurate conclusions, and upon which you can base improvements in practice.

In selecting your instruments, avoid such as will make a display. Gold ferules and pearl handles, set with costly stones, are made for show. As your object is not to impress your patients with terror, it will be better for their nerves, that they should not be awed by a display of costly instruments, *designedly* made conspicuous, until they think your office is intended as a place of torture. In most cases, the patient's imagination has quite as much to do with his suffering, as real pain; and it may be questioned, whether a formidable array of dental instruments is the best anodyne for weak and unstrung nerves.

Your instruments should be of the best, and, like "*Toledo blades*," made for *use.* They should be plain and simple, yet so neat as to attract as little attention as possible.

Your operating case should be well filled, and every thing kept in perfect order. As I have said, your instruments should be of the very best material, the points made with precision, and tempered with the greatest care. Provide yourselves with proper files, and other conveniences; then keep every instrument in perfect order, and ready for use; for even a good workman can not excel, with inferior tools.

Never allow yourselves to be deficient in necessary instruments of any kind; yet, with system and proper judgment, you will need a much less number than you may suppose. Our best operators are, by no means, those who use the greatest number of instruments. Too many will confuse you, while much time will be lost, and your operations may be spoiled, by the saliva, when you are looking for, and exchanging instruments.

It is necessary that you should have a manly independence; and to feel this you must be out of debt. If the kindness of friends, or the forbearance of your teachers, has furnished you with the means of education, or assistance, to enable you to enter upon the discharge of your professional duties, you ought to repay them from your very first earnings. No man has a free will and cheerful heart, who goes to his work the slave of debt. It fetters, at every step of life, and leaves the door open for continual misunderstanding, and may sever the closest friendship. A prompt paymaster must be a good collector. Success in any business depends very much upon a careful attention to bills payable and bills receivable. You will also find that work which is paid for, will fit better, and last longer, than work unpaid for. With some patients, few things stop the uneasiness of a newly filled tooth, or remedy the defects in a new set of teeth, or add to his reputation like the honest payment of the Dentist's bill. Few callings give greater room for misunderstanding than our own, but you will find that honest work for honest people will give but little trouble.

It is a common fault of young practitioners to promise too much. Let me caution you against this; for you will find that *all* are liable to failures. You ought to do your work well, but never *warrant* it. When the real benefits of dentistry were not understood, or acknowledged by the public, perhaps it was proper that the dentist should guarantee a result, which was doubted by a majority of even intelligent people; but now the intelligent regard dental operations as not only useful, but indispensible to health and comfort, and no such necessity for a guarantee now exists. The patients who come to you acknowledge, by their coming, their belief in the benefits of dentistry, and in your skill. You occupy the same relation, in this respect, to your patient, that the physician does to his. The honorable physician never agrees to cure his patient. He knows that if treated ever so well, the patient's own neglect, or carelessness, may make that treatment useless, and even injurious. The *Quack* will not hesitate to guarantee a cure of the most hopeless case, always *provided* he is paid *in advance;* but the consistent physician, honestly tells the probability of success, and then simply pledges his best energies and skill; and on these he relies for business and reputation. No matter how well the Dentist does his work, its durability will depend very much upon the patient's care. If you warrant it, they will be very apt to transfer the responsibility from their own shoulders to yours. The value of your work depends upon the action of both; and if either fail, the work will fail. On your part, the honor of a man, and skill in your profession, call for the very best work. On the part of your patient, care and cleanliness must be observed. You will find it to be of great advantage, especially with children, to request, and *insist*, that they should see you often. You ought then, as far as possible, to insist upon the thorough cleanliness of their teeth; and if they will not attend to this, your work will fail, in spite of the best operations.

If your work does fail, and you can see that the fault is yours, you are bound, as an honest man, to make it good; but if the fault is not yours, your patients have no right to hold you responsible. Our *poorest* and most *unprincipled* dentists are those who give the most positive guarantees against failure.

I think it was Poe who said, "Quack is an ugly word, which never should be used except in the mouths of ducks;" but we have a right to say, that the physician, or dentist, who stoops to the expedient of warranting, in order to gain business, degrades himself and his profession, and *well-merits* the appellation of *Quack.*

Fix your own value upon your services, making it a matter of *pride* and *conscience* to give an equivalent for *fees* demanded and you may confidently rely upon the good judgment of community to sustain and reward you.

In regard to charges for consultation fees, I have only to say that, if your patients take up much of your time, you certainly have the right to charge for it. In this matter, however, you should exercise good judgment. If you err in either direction, let it be on the side of liberality. But an advertisement of "*advice free*," by a physician or dentist, is about as indicative that the advice will be worthless, as that the traveler will find himself enjoying the comforts of a third class hotel, who accepts an invitation to ride in a free 'bus.

Your charges for your services should be in proportion to your skill.

If labor and application has made you more skilled in your profession than your neighbor, there is no reason why you should not receive a just reward. Your skill becomes your *capital*, the value of which no man can estimate who has not passed over the arduous road by which it has been gained.

Were it not for this estimate of skill, the services of the architect or builder would be estimated no higher than those of the wood-sawyer,—the surgeon of Velpeau or Jobert above the bungling of a quack; the ability of a Choat, or a Webster, would not be valued above that of a petifogger.

Our specialty in the healing art will seldom make it necessary for you to visit the sick room; and perhaps never to stand by the bed of the dying. You will, however, be called to witness much suffering; and, when inflicted by yourselves upon sensitive women and children, you will be glad to be relieved from the unpleasant duty. Your patients do not come to you from choice, but from necessity; and you are called to do your duty, even though it conflicts with your sympathy. You should study to make the pain of your operations as light as possible; but no consideration should prevent a faithful performance of your duty. You should always be kind; but there are few cases, where your sympathy expressed, will not excite rather than relieve your patients' fears, and make them less able to endure the necessary suffering.

In no case, be rash or hasty; but when you know your duty, be firm, and push on your operations with manly courage. Your courage will incite courage in your patient; and he will not only submit, but respect you the more, when he sees that a conscienteous desire to benefit him is the mainspring of your action. I am aware that the dentist who does this, will, by some, be called harsh and cruel. The unthinking, who love *humbug*, will prefer the Quack who promises to perform all operations "without pain;" but those who are desirable as patients, will cheerfully submit to whatever you think necessary. It is as true in dentistry as elsewhere, that honesty is the best policy. From my own experience, I am prepared to say, that after explaining to your patients the necessity of thorough manipulation on your part, and a careful attention to the cleanliness of the teeth on their own, if they will not submit and follow your advice, your reputation and pocket will be better off, without their patronage than with it.

You will not please all, and it is better to run the risk of displeasing ignorance and self-will, by doing your duty, than to degrade your profession, and hazard your reputation, by attempting to please unreasonable people.

Great allowance must be made for the lack of judgment and fortitude in children. With these you must be especially careful to unite gentleness with firmness. Never deceive children by the foolish lie that you will not hurt them. If parents have done this, undeceive them, and explain, as far as you can, the real necessity of the case. Your frankness will gain their confidence; and a few moments' talk, in a kind manner, about their sports and plays, will make them feel that you are their friend, and the little ones, in most cases, will sit down to bear the pain with the fortitude of heroes. Do not think you have no time to spend with children in this way, and that it is a matter of no account. The child which is brought to you to-day, will soon meet you on the street as your equal; and if you treat him kindly, he will remember you, and prove your true friend.

Sometimes no talking will induce children to submit to what you think necessary. In such cases, if your services are of *real* importance to them, you should not hesitate to resort to force. This should always be done in a kind, but resolute manner, and when you undertake it, never yield, until you have accomplished your object.

You should strive to do all work well, and never degrade a noble calling by the dishonest expedients of quacks. You will certainly meet with failures in your operations, and sometimes when you least expect it. Business may come in slowly, and dark hours seem to gather around you; for "hope deferred maketh the heart sick." But if you are patient in gaining work, patient in doing it well, and are honest and faithful, you need not be discouraged; for though the school of experience gives some hard lessons, patient striving will lead to success.

You must aim to have an unsullied character. The dentist has the most intimate relation with his patients; and he owes it to himself and others that his thonghts and example should be elevating. You can not separate your private, from your professional character. The habitual Sabbath breaker, the irreverent scoffer, the foul blasphemer, or the sensualist, who dishonors God, and does violence to the most sacred feelings of the human heart, does not deserve a place in any honorable profession.

A few years since, the advantages which you have enjoyed, could not be procured at any cost. In fact, the demand for dentistry was then small; and it required very little natural or acquired ability to meet all demands for the dentist's skill. No dentist was suspected of being a scientific man. A few months' teaching was enough to enable almost any one to become a dentist; or an ingenious Yankee could "take it up of his own head," without the risk of being accused of having an excess of brains.

As refinement and civilization increased, the demand for dental operations, dentists increased in number and character. These men saw that dentistry could be made a branch of the healing art,—a specialty in medicine. They determined that they would withhold no secret from their professional brethren, who were prepared to engage in this enterprise with them. They formed the "American Society of Dental Surgeons," where they might freely consult together upon the principles and practice of their profession. The "American Journal of Dental Science" was commenced, with its pages open to all who had any thing to communicate of advantage to the new profession. It was soon found that the improvements in the art had been so rapid and so great, that a few months' private instruction was not enough to qualify beginners to practice it. A thorough and systematic course of preparatory training was demanded; and to meet this requirement, the Baltimore college of Dental Surgery was started. A few years later the Ohio College, and still later the Pennsylvania and New York Colleges, the latter of which perished in its infancy, not on account of its "*feeble* progeny," but from fire and treachery.

Discovery has followed discovery,—art and scientific research have been stimulated, and applied in our art, so that dentistry has risen, in the last quarter of a century, from a catch-penny calling, to a useful and respectable profession. Some of its pioneers have lived to witness these great triumphs. To-day they see it enjoying the benefits of colleges, and a literature, of which, professions much older might well be proud, and men engaged in practice, whose abilities would be ornaments to *any* profession.

As you look about you and see the changes that have been made in our art, you must not imagine that the world has stood still to witness the progress of our Profession. Our calling has but partaken of the spirit of the age. In the honored field of medicine, in minerology, in astronomy, in geology, in mechanical invention, in agriculture, and in almost every other field of labor, there has been a great improvement. We are now living in the most remarkable age of the world. Every avenue to wealth and eminence is crowded; and every one is striving with his fellow, for distinction. Into this world of thought and labor you are about to enter; and you owe it to this College, that, like dutiful sons, you shall win laurels, to lay at the feet of your *Alma Mater*.

Aside from your own character, which must share the reputation of your profession, you should seek to fill its ranks with educated men. It is doing a great injury to ourselves when we take the young men from the plow, the work-shop, or the counting-room, and, after a few months spent in our offices, send them forth to practice dentistry. The time has come when we should rely upon our dental colleges for the training of those who desire to enter our profession in a legitimate way. Deny it as much as some will, our colleges offer advantages for training the student, possessed by *no private office.* Private instruction in ours, as in the medical profession, is desirable; but it should only be given to students preparatory to college instruction.

There is no common trade which admits men to its ranks without discipline and training. Six months, or a year, with a shoe-maker, may make a cobler, but not a finished workman. You would not trust the apprentice of six months to shoe your horse. You ask that the tailor shall be master of his work; and yet it is the custom of many dentists (and some from whom we should expect better things), to take young men, and those who can hardly get a living at any thing else, and, after from six to eighteen months' drudgery in the office, send them out with letters of recommendation, to defraud the public, and to degrade our calling.

In every profession which requires skill, there is great need of thorough training; and yet Dentistry, which requires a knowledge of anatomy, chemistry, physiology, pathology, materia medica, therapeutics, and mechanics and the arts, is too often left to a few months' careless practice, or indifferent instruction, given in the intervals of business. Our profession has not, as yet, given sufficient encouragement to Dental Colleges. Many of them are apt to speak of them as unnecessary. The same was once true of Medical Colleges; but in all civilized communities, they are more regarded as necessary for a thorough medical education.

We have learned to call those quacks who attempt to practice medicine without having passed through the discipline of a Medical College; and may the day soon come, when the community shall acknowledge, by the bitterness of experience, that it has learned the same wholesome lesson, in regard to our colleges and profession. I am aware that many, who have not enjoyed this discipline, have, by their perseverance, worked themselves up to an enviable distinction, both in the medical and dental professions; but this only proves that those men possessed minds and energy sufficient to grapple with and overcome great difficulties. Many of them commenced when we had no dental colleges, and they have well earned their enviable distinction.

The necessity no longer exists for thus entering our profession. The demand of the age is for the best; and he who hopes for success must come to his calling thoroughly prepared. Without this the lawyer is a pettifogger, the craftsman a tinker, the doctor a quack, and need I add, that the man who writes "Dentist" after his name, without a thorough knowledge of his calling, combines them all,—a pettifogger, tinker and quack.

Gentlemen, you owe it to yourselves to take the highest ground for the honor of your profession; and as it advances in scientific acquirements, you will find the day will come when our Dental Colleges will be regarded as necessary for the accomplished dentist, as the medical colleges are for the skilled in medicine, or as universities, like Harvard or Yale, are for the finished scholar.

With energy, sparkling with the sunlight of hope, you look out upon the world, from these pent up halls, and see a wide field for usefulness inviting you forth to labor, and in the imagination, so natural to youth, you see reputation, wealth and position joining hands, to bid you welcome, and crown your efforts with success; but here let me remind you, that wealth never abides with the prodigal, reputation never comes to the profligate, nor fame to the sluggard.

You should husband well your time, and remember that life and reputation are made up of little things; that every act adds to or detracts from an honored name. You can not expect to gather figs of thorns, or grapes of thistles. Character is not a thing of chance, nor skill the reward of ignorance or idleness.

"Link by link the chain is made;
Pearl by pearl the costly braide;
The daily thread of hopes and fears
Weave up the woof of many years.
And well thy labors shall have sped
If well thou weavest the daily thread."

It remains but for you to say farewell to these scenes of pleasure and distinction. As you go hence, to make the trial of the hard realities of life, you should remember that it is not all of life to live. He who estimates life by the money he hoards up, or the number of years, lives but in vain.

"We live in deeds, not years, in thoughts not breath,
In feelings, not in figures on a dial;
We should count life by heart-throbs—he most lives
Who thinks most—feels the noblest—acts the best."

Gentlemen,—in behalf of my profession, I bid you welcome as laborers in its ranks. In behalf of the officers of the Ohio College of Dental Surgery, whose partiality has conferred upon me the honor of addressing you on this occasion, I bid you a hearty God-speed, assuring you that if faithful and honest, you will find a noble field of labor, which will generously reward you for every toil.

